# Comparative analysis of CNN, Transformer, and Mamba-based models for automated eye disease classification from fundus images

**DOI:** 10.1371/journal.pone.0360171

**Published:** 2026-10-26

**Authors:** Md. Ahanaf Arif Khan, Sangeeta Biswas

**Affiliations:** Department of Computer Science and Engineering, Faculty of Engineering, University of Rajshahi, Rajshahi, Bangladesh; Zhejiang University, CHINA

## Abstract

We present a comparative analysis of six models belonging to Convolutional Neural Network (CNN), Transformer, and Mamba families for automated eye disease classification from fundus images under identical experimental conditions. To the best of our knowledge, our work is the first to explore Mamba-based models for automated multi-class eye disease classification from color fundus images. Our analysis suggests that, when evaluated fairly, modern CNN, Transformer, and Mamba-based models provide similar performance for eye disease classification on the Eye Disease Image Dataset (EDID). Along with analyzing individual model performance, we further evaluate performance using a logit-level ensemble of the top three models. The ensemble results imply that these model families capture partially complementary visual features, resulting in a slight performance gain when combined. In both cases, class-wise performance examination indicates that diseases with distinctive structural characteristics are recognized more consistently, while categories with subtle or overlapping features remain challenging. Our implementation, including the training and evaluation pipelines as well as the data splits, is publicly available at https://github.com/ahanaf019/EyeDisease2026.

## Introduction

The human eye is a highly complex and sensitive organ that enables us to visualize the world around us, and its health is crucial to the overall quality of life. When the eye is affected by disease, it can cause significant problems in our vision [1,2] and eye-based biometric authentication [3,4]. Many eye diseases begin early in life and become more severe and complex with age [1]. Early and accurate identification of these diseases is essential to preserve visual function. Fundus imaging serves as a cornerstone of eye disease diagnosis by providing a non-invasive and cost-effective means of visualizing retinal vasculature, the optic disc, and macular structures. However, manual analysis of fundus images requires substantial time, even for clinical experts. This challenge is particularly acute in resource-limited regions with large populations, where the scarcity of trained eye specialists restricts access to timely eye care. As a result, automated eye disease classification systems are essential in these cases to provide scalable and reliable diagnostic support.

To support the development and evaluation of automated eye disease classification systems, several publicly available fundus image datasets have been introduced in recent years [5–10]. Among them, the Eye Disease Image Dataset (EDID) [10,11] provides a diverse collection of labeled ocular images covering nine diseases, such as Central Serous Chorioretinopathy (CSCR), Diabetic Retinopathy (DR), Optic Disc Edema (ODE), Glaucoma (GLC), Macular Scar (MS), Myopia (MYA), Retinal Detachment (RD), Retinitis Pigmentosa (RP), and Pterygium. Therefore, this dataset has the potential to serve as a valuable benchmark for comparative studies. However, the EDID remains relatively underexplored. Existing studies on this dataset use varied deep neural network architectures, training protocols, and dataset splits, which makes direct comparisons and reproducibility challenging [12–17]. Establishing a controlled and uniform experimental setting is, therefore, crucial for drawing meaningful conclusions about the relative strengths of modern deep neural networks for fundus image-based eye disease classification.

To address these issues, this study performs a controlled comparative analysis of Convolutional Neural Network (CNN), Transformer [18,19], and Mamba [20,21]-based models for automated multi-class eye disease classification from color fundus images using the EDID, under identical experimental conditions. While CNN- and Transformer-based models have been explored in this domain, Mamba-based models have not received any attention for automated eye disease classification, despite their recent success in other vision tasks. We adopt a unified pipeline for preprocessing, model training, and evaluation to ensure methodological fairness. In this setting, we systematically evaluate two representative architectures from each model family to compare their relative performance. Additionally, we construct a logit-level ensemble of the top-performing models to investigate whether combining diverse architectural paradigms yields complementary performance gains. Through this design, our work aims to provide a fair and transparent empirical comparison that clarifies how architectural choices influence performance under carefully controlled experimental conditions.

### Recent works

Recent work in automated eye disease classification using the EDID has explored CNNs, transformers, and their hybrid models to capture both local and global features from fundus images. This dataset does not provide any specific training, validation, or test sets. Therefore, each previous study used its own data partitioning approach. Many studies emphasize lightweight design, handling of class imbalance, and clinically relevant metrics. However, differences in preprocessing, augmentation, and, especially, data-splitting protocols have led to substantial variation in performance. Rieck et al. [15] proposed a hybrid architecture combining EfficientNet-B4 with Swin Transformer V2 (SwinV2) to capture both local details and global pathological context. They used 80% of the data for training and 20% for testing. They evaluated their model with stratified 5-fold cross-validation. Their model achieved an overall accuracy of 0.7640 and a high true negative rate of 0.9560. It had particularly strong performance for DR and RP, though the performance of GLC was lower. Sevik et al. [14] manually curated 1,841 images from EDID by excluding duplicate, poor-quality, incorrectly labeled, and unclear images. They applied stratified data splitting and targeted augmentation to mitigate class imbalance. They evaluated three pretrained architectures, with a YOLOv11-based classifier demonstrating superior performance. Their final model achieved an overall accuracy of 0.8610 on the test set.

In some cases, data leakage can make reported results appear better than they are. Note that data leakage occurs when data from the test or validation sets are inadvertently included in the training set, leading to overly optimistic and misleading performance estimates. Santoso et al. [16] presented a lightweight deep learning approach for nine-class disease classification from fundus images. They reported strong performance from EfficientViT, which had only three million parameters. However, the reliability of these results is compromised by a major methodological flaw: the model was trained on augmented versions of the original images. The original images were used for validation and testing. This resulted in data leakage, whereby the model was effectively evaluated on images it had already seen, which likely inflated performance metrics and does not reflect true generalization. Rieck et al. [13] proposed an approach that emphasized tailored preprocessing rather than architectural complexity. They applied Gaussian filtering to reduce noise and enhance fine structures before using a ResNet-50 backbone. They utilized a 5-fold cross-validation approach. However, data leakage occurred because cross-validation was applied on the augmented images of EDID. This prevents a true assessment of the model’s generalization ability and limits confidence in its performance on genuinely unseen data. Arnob et al. [12] proposed a lightweight, attention-augmented CNN. Their method utilized SMOTE-based oversampling and extensive augmentation. They achieved a test accuracy of 0.8790. Although they report strong true-positive rates across most classes, their reported performance is subject to data leakage because SMOTE-based oversampling was applied prior to data splitting. This may have inflated the results and does not reflect the model’s true performance. Gu et al. [17] proposed a Transformer-based architecture named SwinECAT. Their model was designed to better capture both localized retinal lesions and global contextual dependencies while maintaining parameter efficiency. Their model achieved an accuracy of 0.8829 on the test set. However, their analysis was also affected by data leakage since they utilized the augmented images for training, validation, and testing.

The issues in most prior work highlight the importance of conducting evaluations under controlled, transparent, and reproducible experimental conditions when comparing models. Consistent data partitioning and unified training protocols are essential to ensure fair comparisons across architectural paradigms, reduce the risk of data leakage, and yield more reliable performance estimates. These factors are crucial for drawing meaningful conclusions about the relative strengths of diverse deep learning approaches and for supporting the development of clinically dependable diagnostic systems.

### Description of Eye Disease Image Dataset (EDID)

EDID [10,11] contains a total of 5,335 original images. The dataset also provides 16,242 augmented images, produced by applying rotation, width and height shifting, translation, flipping, and zooming to the original images. It contains two types of images: color fundus images and anterior segment images. These images were collected over eight months from two hospitals in Faridpur, Bangladesh. The images were captured using Topcon TL-211 and Topcon TRC-50DX fundus cameras coupled with Nikon DSLR cameras. All images were resized to a uniform resolution of 2004×1690. The fundus images are categorized into nine classes: Healthy, CSCR, DR, ODE, GLC, MS, MYA, RD, and RP, while the anterior segment images belong to a single class, Pterygium. Fig 1 illustrates three randomly selected examples from the nine fundus image categories in the dataset.

**Fig 1:**
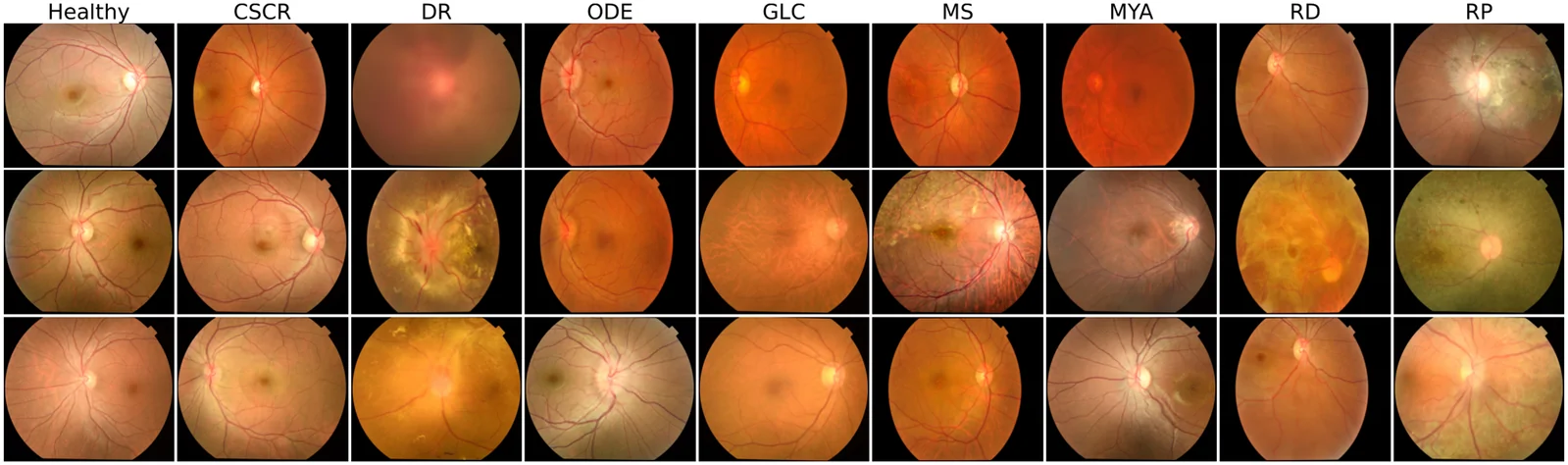
Representative fundus images from the EDID. Each cell corresponds to one of the nine fundus image categories (Healthy, CSCR, DR, ODE, GLC, MS, MYA, RD, and RP) with three example images per category.

A healthy eye is defined as one exhibiting normal retinal structure and function, without evidence of disease, pathology, or degenerative changes. CSCR is a retinal disorder causing macular detachment, often linked to retinal pigment epithelial dysfunction and choroidal changes [22,23]. It mainly affects men aged 20–50 and causes blurred vision, central scotoma, or visual distortion. In DR, high blood sugar causes blood and fluids to leak from the retina’s blood vessels [24]. ODE is the swelling of the optic disc due to the accumulation of fluid or axonal distension, which can result from various causes, including elevated intracranial pressure, inflammation, axoplasmic stasis, or structural optic nerve abnormalities [25]. GLC results from progressive damage to retinal ganglion cells, commonly associated with elevated intraocular pressure, leading to optic nerve degeneration and characteristic cupping of the optic disc [26]. MS develops primarily due to damage to the retinal pigment epithelium and an abnormal inflammatory response leading to geographic atrophy [10]. MYA is a refractive error causing blurred distance vision that can usually be corrected with glasses, contact lenses, or surgery. High myopia and pathological myopia significantly increase the risk of serious sight-threatening complications that can lead to permanent vision loss [27]. RD is a sight-threatening eye disease characterized by the separation of the neurosensory retina from the underlying retinal pigment epithelium [28]. RP is a hereditary retinal disorder marked by the gradual degeneration of rod and cone photoreceptors, leading to night blindness during adolescence, peripheral vision loss in early adulthood, and eventual impairment of central vision [29,30].

In our experiments, we utilized the original images, as the augmented dataset contains multiple transformed copies of each original image. We also excluded anterior segment images from the dataset for two reasons. First, this category contains only 17 images in the original dataset, making it highly underrepresented. Second, the pronounced visual differences between anterior segment and fundus images would allow the models to identify this class based primarily on image modality rather than disease-related features, even when no pathological evidence is present. After excluding these images, we were left with 5,318 original fundus images. We used the whole dataset without considering image and label quality. We partitioned the dataset into training, validation, and test sets using a stratified 70-10-20% split to preserve class distributions. A fixed random seed (224) was used to ensure reproducibility. Using scikit-learn, the split was performed once at the file level, before oversampling or data augmentation, to prevent data leakage. To assess the risk of image-level leakage across splits, we performed a full pairwise near-duplicate check using Structural Similarity Index Measure (SSIM) [31] (threshold ≥0.98) across all 2,824,276 class-bucketed image pairs among the 5,318 original fundus images. We identified 152 near-duplicate pairs, of which 71 crossed split boundaries. We did not remove these 71 pairs because they did not alter the basis of our comparative evaluation. All six models were trained and evaluated on the identical data split, and therefore the same potentially overlapping samples were present in the evaluation set for every model. Consequently, no individual model received an advantage from this overlap relative to the others, and the relative performance comparisons among the six models remain valid. This situation also differs from the systematic leakage reported in prior EDID studies [12,13,16,17], where models were trained directly on augmented or oversampled versions of validation/test images.

To address class imbalance in the training set, classes containing fewer than 500 images were oversampled by replication to reach 500 samples per class. Oversampling by replication was chosen to keep our methodology simple and transparent. More advanced methods, such as GANs and diffusion models, were not chosen because they typically require large amounts of training data, which were not available for several minority classes in this study (e.g., CSCR, ODE, MS, MYA, RD, and RP). In addition, synthetically generated retinal images may not reliably preserve clinically relevant anatomical and pathological features. To reduce overfitting despite replication, extensive on-the-fly augmentation was applied during training, ensuring that the model rarely encountered the exact same version of an image twice. The distribution of images across the training, validation, and test sets, along with the results after oversampling, is summarized in Table 1.

**Table 1:** Distribution of fundus images across training, validation, and test sets.

Class	Total	Train Set (70%)	Val Set (10%)	Test Set (20%)	Oversampled Train Set
Healthy	1,024	717	103	204	717
CSCR	101	71	10	20	500
DR	1,509	1,056	152	301	1,056
ODE	127	89	13	25	500
GLC	1,349	944	135	270	944
MS	444	311	44	89	500
MYA	500	350	50	100	500
RD	125	87	13	25	500
RP	139	97	14	28	500
Total	**5,318**	**3,722**	**534**	**1,062**	**5,717**

The final column reports the number of samples per class in the oversampled training set.

### Models explored in this study

We constructed models using six backbones belonging to three architectural families: CNN-based models (RegNetY [32] and ConvNeXt [33]), Transformer-based models (Data-Efficient Image Transformer (DeiT) [34] and SwinV2 [35]), and Mamba-based models (Vision Mamba (Vim) [36] and MambaVision [37]). We chose these backbones because they are widely adopted across diverse vision tasks, were introduced from 2020 onward, achieve strong ImageNet-1k top-1 accuracy as a proxy for general representational capability, and reflect substantially different design philosophies within each architectural family rather than closely related variants of the same approach. For CNNs, RegNetY represents a classical inductive-bias-heavy design with structured width progression and channel-wise squeeze-and-excitation attention, while ConvNeXt represents a modernized CNN that incorporates Transformer-inspired design choices like large kernels, depthwise convolutions, and layer normalization, while preserving spatial locality. This pairing allows us to contrast different convolutional feature extraction approaches within the same family. For Transformers, DeiT follows a non-hierarchical design where global self-attention is applied uniformly across all layers at a fixed resolution, while SwinV2 uses a hierarchical, window-partitioned design that introduces spatial locality back into the attention mechanism. This contrast tests whether global versus local-to-global attention strategies differ meaningfully for retinal image analysis. For Mamba-based models, Vim is a pure SSM architecture adapted for vision through bidirectional scanning, maintaining a non-hierarchical structure, while MambaVision-B is a hybrid architecture that combines convolutional blocks, Mamba blocks, and self-attention within a hierarchical design. Together, these models allow us to compare a pure SSM-based design with a hybrid approach that combines state-space modeling, convolutions, and attention mechanisms.

To ensure a fair comparison, we selected models with comparable parameter counts. In this regard, we selected the base (B) variants of all models, except RegNetY-16GF. RegNetY-16GF was selected as the closest RegNetY variant to the base configurations of other models since no ‘base’ variant exists in the RegNetY family, making 16GF the most appropriate size-matched choice. Table 2 summarizes the parameter sizes and ImageNet-1k benchmark performance of the evaluated backbones. Figs 2A, 2B, 2D, 2G, 2E, and 2H illustrate the overall architectures of the RegNetY-16GF, ConvNeXt-B, DeiT-B, SwinV2-B, Vim-B, and MambaVision-B backbones, respectively, highlighting their main structural components and design differences. We briefly describe each model below.

**Table 2:** Comparison of model size and ImageNet-1k top-1 accuracy for the six different backbone architectures.

Type	Backbone	Year	Params (M)	Acc (top-1)
CNN	RegNetY-16GF [32]	2020	83.6	80.4
	ConvNeXt-B [33]	2022	88.6	83.8
Transformer	DeiT-B [34]	2021	86.6	81.8
	SwinV2-B [35]	2022	87.9	84.6
Mamba	Vim-B [36]	2024	98.0	83.2
	MambaVision-B [37]	2025	97.7	84.2

**Fig 2:**
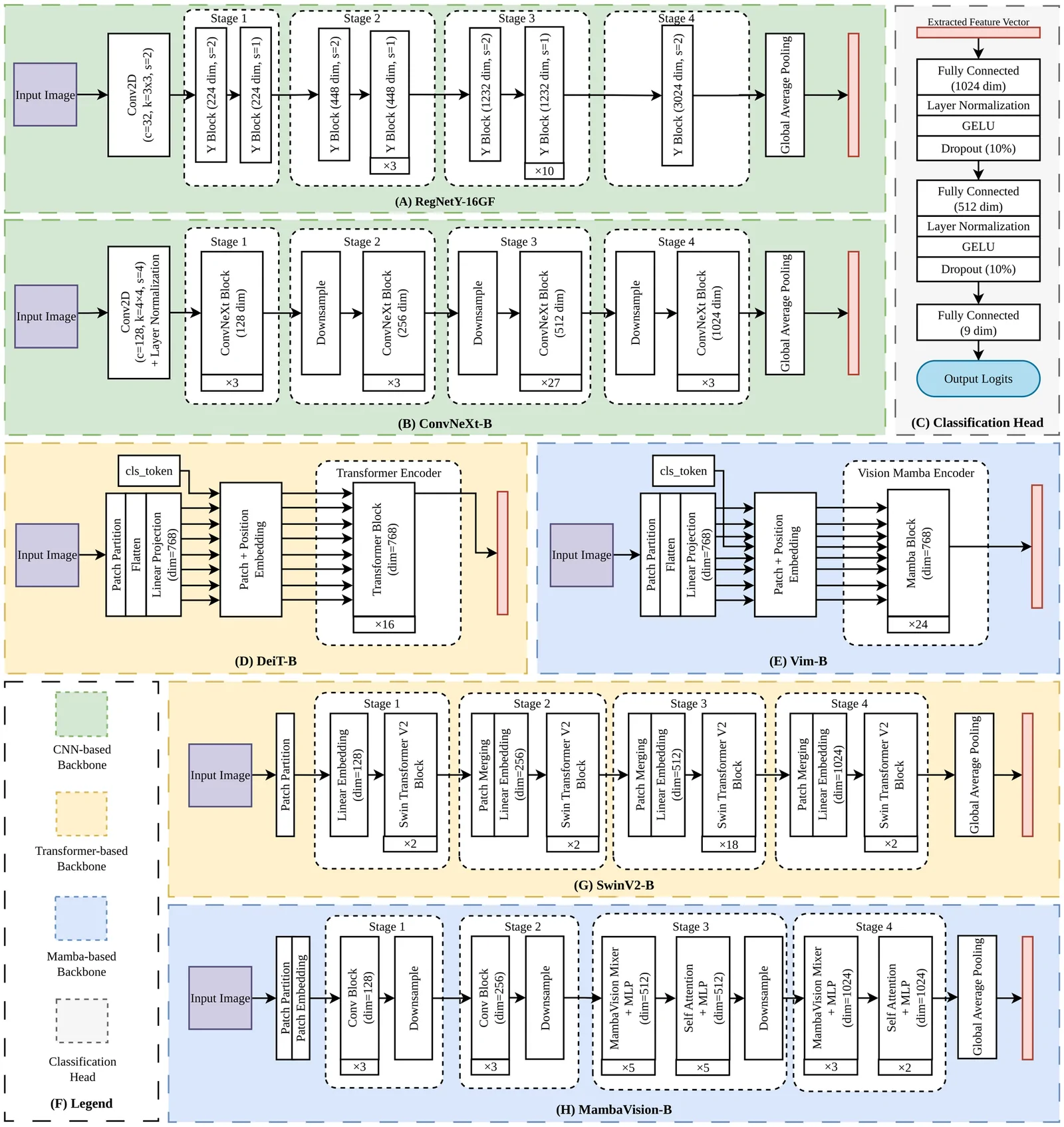
Overall architecture of the six backbone model architectures and the classification head. Subfigures (A), (B), (D), (G), (E), and (H) show the RegNetY-16GF, ConvNeXt-B, DeiT-B, SwinV2-B, Vim-B, and MambaVision-B models, respectively, illustrating their key structural components and stage-wise design. Convolutional, Transformer-based, and Mamba-based models are represented using different background colors. Subfigure (C) shows the shared classification head composed of fully connected layers with normalization, GELU activation, and dropout, which maps the extracted feature embeddings to final class logits, and subfigure (F) shows the legend.

### Convolutional Neural Network (CNN)

#### RegNetY.

RegNetY [32] is a CNN derived from the RegNet design paradigm, which formulates network widths as simple parametric functions rather than relying on handcrafted configurations. The architecture follows a hierarchical staged design, where each stage *i* contains $d_{i}$ blocks with width $w_{i}$, determined by a linear parameterization of the form


$$\begin{array}{c} w_{i}=w_{0}+w_{a}\cdot i, \end{array}$$

where *w*_0_ represents the initial width and $w_{a}$ denotes the width slope. RegNetY incorporates the Squeeze-and-Excitation (SE) mechanism in its blocks to enhance channel-wise feature recalibration, which computes channel attention as


$$\begin{array}{c} 𝐬=\sigma \left(W_{2}\cdot \delta (W_{1}\cdot \text{GAP}(𝐗))\right), \end{array}$$

where GAP denotes global average pooling, δ is a ReLU activation, and σ is the sigmoid function. RegNetY thus represents the classical convolutional design philosophy in its most committed form: small local kernels, structured channel scaling, hard-coded spatial inductive biases, and explicit channel-wise recalibration as the sole mechanism for global information aggregation. For our analysis, we adopt the RegNetY-16GF configuration, whose architecture is depicted in Fig 2A.

#### ConvNeXt.

ConvNeXt [33] starts from the same convolutional foundation as RegNetY but systematically modernizes it by importing design decisions from the Transformer architecture [18]. Its basic block applies a large-kernel depthwise convolution followed by pointwise layers:


$$\begin{array}{c} 𝐘=𝐗+W_{2}\cdot \text{GELU}(\text{LN}(\text{DWConv}(𝐗))\cdot W_{1}), \end{array}$$

where DWConv uses a 7×7 kernel, LN denotes layer normalization, and *W*_1_, *W*_2_ are pointwise convolutions. Compared to RegNetY, the structural differences are substantial: the receptive field per block is significantly wider, batch normalization is replaced by layer normalization, there are fewer activations and normalization layers, and the SE-based channel recalibration mechanism is dropped entirely in favor of the inverted bottleneck structure. This results in a model that retains the locality and translation equivariance of convolution while aligning its optimization landscape much more closely with that of Transformers. Where RegNetY commits fully to the classical convolutional inductive bias, ConvNeXt interrogates how far that bias can be relaxed before the architecture ceases to be convolutional in any meaningful sense. We utilized the base configuration, ConvNeXt-B, in our analysis, whose architecture is shown in Fig 2B.

### Transformer

#### Data-Efficient Image Transformer (DeiT).

DeiT [34] builds upon the Vision Transformers (ViTs) [19] architecture, which departs from the convolutional paradigm entirely. Rather than building context through successive local operations like in CNNs, ViT divides the image into fixed-size patches, linearly projects each into an embedding space, and processes the resulting sequence $\left\{{𝐱}_{1},...,{𝐱}_{N}\right\}$ through stacked multi-head self-attention layers. A learnable class token **x**_cls_ is prepended to aggregate global information for classification. The core mechanism is the multi-head self-attention layer, which enables the model to capture global dependencies across all image patches. The self-attention operation is computed as:


$$\begin{array}{c} \text{Attention}(𝐐,𝐊,𝐕)=\text{softmax}\left(\frac{𝐐{𝐊}^{⊤}}{\sqrt{d}}\right)𝐕, \end{array}$$

where **Q**, **K**, and **V** are the query, key, and value matrices and *d* is the key dimension. Every token attends to every other token at every layer, with no positional or hierarchical prior constraining which interactions are permitted. The architecture is entirely flat: spatial resolution and embedding dimension remain fixed throughout all layers, and no multi-scale representation is constructed. It enables rich feature interactions across the entire image without the locality constraints inherent in CNNs. DeiT presents an improved training strategy for ViTs incorporating knowledge distillation. This strategy significantly reduces dependence on large-scale pretraining datasets and achieves enhanced performance compared to the original ViT. We used the base configuration, DeiT-B, in our analysis; its architecture is illustrated in Fig 2D.

#### Swin Transformer V2 (SwinV2).

SwinV2 [35] accepts the Transformer’s self-attention mechanism as its core operation but reintroduces structural priors that DeiT deliberately discards. SwinV2 extends the original Swin Transformer [38] by introducing scaled cosine attention and continuous relative position bias in the attention formulation. Rather than computing attention globally like in DeiT, SwinV2 restricts it to local windows, reducing complexity from *O*(*N*^2^) to *O*(*NM*) where *N* is the number of patches and *M* is the window size. Cross-window interactions emerge through a shifted window strategy applied across alternating layers. The attention is formulated as:


$$\begin{array}{c} \text{Attention}(𝐐,𝐊,𝐕)=\text{softmax}\left(\frac{\cos(𝐐,𝐊)}{\tau }+B\right)𝐕, \end{array}$$

where cos(𝐐,𝐊) denotes the cosine similarity between **Q** and **K**, τ is a learnable temperature parameter and *B* is the continuous relative position bias. Unlike DeiT, which treats all spatial positions equivalently regardless of their distance, SwinV2 explicitly encodes metric spatial relationships between tokens and builds representations hierarchically across four stages of progressively reduced resolution. This local-to-global progression of contextual aggregation mirrors the multi-scale design of CNNs, but achieves it through windowed attention rather than convolution. The contrast with DeiT is therefore not merely one of efficiency; it reflects a deeper disagreement about whether spatial locality and hierarchy should be imposed as architectural constraints or left to be learned freely from data. We utilized the base configuration, SwinV2-B, whose architecture is illustrated in Fig 2G.

### Mamba

#### Vision Mamba (Vim).

Unlike CNNs and Transformers, which rely on convolution and self-attention, respectively, Vim [36] utilizes a third distinct mechanism, namely Mamba, which is based on State Space Models (SSMs) [21] for spatial context aggregation. The foundation of Vim rests upon Structured SSM (S4) [20]. Rather than computing pairwise token interactions as in Transformers, or composing local filters as in CNNs, Vim propagates context through a hidden state that is updated recurrently at each position along a scan path:


$$\begin{array}{c} {𝐡}_{t}=\bar{𝐀}{𝐡}_{t-1}+\bar{𝐁}{𝐱}_{t},\;{𝐲}_{t}=𝐂{𝐡}_{t}, \end{array}$$

where ${𝐱}_{t}$ is the input token at position *t*, ${𝐡}_{t}$ is the hidden state, and $\bar{𝐀}$, $\bar{𝐁}$, **C** are learned parame*t*ers. Vim introduces a selective scan mechanism which makes these parameters input-dependent, allowing the model to modulate how much information is retained or discarded at each step. To adapt this inherently sequential model to two-dimensional images, Vim tokenizes the image into patches and processes them through a bidirectional scan, merging forward and backward representations to reduce sensitivity to scan order. Each Vim block applies this bidirectional Mamba module with a residual connection:


$$\begin{array}{c} {𝐱}_{\ell }={Mamba}_{\text{bi}}(LN({𝐱}_{\ell -1}))+{𝐱}_{\ell -1}. \end{array}$$

where ${𝐱}_{\ell -1}$ and ${𝐱}_{\ell }$ denote the input and output feature representations of the ℓ-th layer, respectively; ${Mamba}_{\text{bi}}(\cdot )$ denotes the bidirectional Mamba block; and the additive term ${𝐱}_{\ell -1}$ corresponds to a residual connection. Like DeiT, the architecture is non-hierarchical and maintains a fixed resolution throughout. However, unlike DeiT, its global context is represented implicitly and compressed into a fixed-size hidden state, rather than being computed through explicit pairwise interactions, resulting in linear rather than quadratic complexity with respect to sequence length. In our experiments, we utilized the base variant, Vim-B, whose architecture is illustrated in Fig 2E.

#### MambaVision.

MambaVision [37] takes the SSM mechanism of Vim as a starting point but embeds it within a substantially richer architectural framework. Where Vim applies bidirectional scanning directly to raw patch embeddings in a flat, uniform design, MambaVision first processes the image through two stages of CNN-based residual blocks, producing spatially coherent feature representations before any state-space modeling is performed. State-space modeling is then applied in Stages 3 and 4 through the MambaVision mixer, which employs a dual-branch architecture. One branch applies a selective scan for sequential context modeling while the other applies convolution and activation to preserve spatial structure, with both branches projecting to $\frac{C}{2}$ dimensions before being concatenated and projected back to *C*. Each layer in these stages follows:


$$\begin{array}{c} {\hat{𝐱}}_{n}=\text{Mixer}(\text{LN}({𝐱}_{n-1}))+{𝐱}_{n-1},\;{𝐱}_{n}=\text{MLP}(\text{LN}({\hat{𝐱}}_{n}))+{\hat{𝐱}}_{n}, \end{array}$$

where Mixer alternates between MambaVision blocks in the first $\frac{N}{2}$ layers and self-attention blocks in the final $\frac{N}{2}$ layers, allowing the model to recover explicit global context after the sequential state-space modeling operation. We utilized the base variant, MambaVision-B, for our experiments, its architecture is illustrated in Fig 2H.

## Experimental setup

### Model construction

All backbone models used in this study (see Table 2) were pre-trained on the ImageNet-1k dataset. To ensure a fair and controlled comparison across different architectures, we replaced each backbone’s original classification head with our customized classification head. Using a consistent head design across all models prevents performance differences from being influenced by variations in classifier architecture, allowing the evaluation to focus primarily on the representational capabilities of the backbones themselves. This design choice is particularly important because classification head design involves many architectural design choices, including layer depth, neuron count, activation functions, normalization methods, pooling strategies, and regularization techniques. Tuning these components separately for each backbone would substantially increase experimental complexity and introduce additional sources of variation, making it difficult to attribute performance differences solely to the backbone architectures.

Our classification head consists of three fully connected layers. It first projects the input features to a 1024-dimensional embedding space using a fully connected layer, followed by Layer Normalization, a GELU activation, and dropout. The representation is then projected to 512 dimensions via a second linear layer, followed by Layer Normalization, GELU, and dropout. Finally, a fully connected layer maps the features to the output dimension, producing the classification logits. The structure of our classification head is shown in Fig 2C.

We also constructed an ensemble of the top three models via logit-level fusion. We used two fusion methods: mean and max. Mean fusion aggregates the predictions by computing the average of the logits produced by each individual model, resulting in a consensus prediction that reflects all models equally. In contrast, max fusion selects the maximum logit across models for each class, thereby emphasizing the most confident prediction among ensemble members.

### Training setup

All of our models were trained using the same hyperparameter configuration. The models were trained for 50 epochs with a batch size of 32. The model parameters were optimized using the AdamW optimizer with a learning rate of $1\times 10^{-4}$ and a weight decay of 0.01 to prevent overfitting. Gradient clipping with a maximum norm of 1.0 was applied, and Exponential Moving Average (EMA) with a decay rate (α=0.999) was employed for stable and reliable training. The model was optimized using the cross-entropy loss function. The learning rate followed a cosine annealing schedule with a minimum learning rate of $1\times 10^{-6}$. Validation was performed at the end of each epoch, and the model with the lowest validation loss was saved as the best-performing checkpoint.

### Preprocessing and augmentation

All images were first resized to 256×256 pixels. During training, a random resized crop of 224×224 was extracted. Afterward, augmentations were applied to improve generalization and robustness. The training augmentations included spatial transformations such as horizontal flipping, rotation, translation, and shearing, as well as photometric transformations including illumination and contrast enhancement using CLAHE, brightness–contrast adjustment, and mild color jittering. For CLAHE, the clip limit was sampled between 2.0 and 4.0. We also employed random image sharpening, embossing, Gaussian noise, and JPEG compression artifacts. These augmentations encouraged the models to rely on semantically meaningful features rather than spurious visual cues, thereby improving generalization.

During evaluation, a 224×224 center crop was extracted from the resized image. Contrast was enhanced using CLAHE with a fixed clip limit of 3.0 to improve local detail visibility for more reliable model assessment. This clip limit was selected as the midpoint of the training range [2.0, 4.0] and was fixed a priori without tuning on the validation set. In both training and evaluation, pixel values were scaled to the [0,1] range and normalized channel-wise using the mean and standard deviation of the ImageNet-1k dataset, ensuring consistent input distributions across all models.

### Hardware and software configuration

All experiments were conducted on a system equipped with an Intel Core i5 13400F CPU, 32GB DDR4 memory, and a single Nvidia GeForce RTX 3090 GPU with 24GB VRAM. The system ran Ubuntu 22.04 LTS with Python 3.10. We used PyTorch 2.7.1 as the deep learning framework, along with other Python-based libraries such as torchvision, torch_metrics, torch_ema, timm, transformers, mamba_ssm, numpy, pandas, matplotlib, ptflops, scikit-learn, and opencv-python, to conduct our experiments.

### Evaluation metrics

We compared model performance using micro-averaged accuracy, macro-averaged accuracy, macro-averaged F1 score, and macro-averaged one-vs-rest Area Under the Receiver Operating Characteristic Curve (AUC-ROC). All metrics were computed using the torchmetrics library (version 1.8.2). Additionally, we computed class-wise accuracy, precision, and F1 score to assess model performance in each class. We also computed confusion matrices to gain detailed insight into the models’ classification performance.

We evaluated computational efficiency on an NVIDIA RTX 3090 GPU using FP32 precision. Peak GPU memory and training time per epoch were measured during training, with peak memory recorded over an initial warm-up epoch and training time averaged over three subsequent epochs. Inference throughput (FPS) was measured on the validation set with a batch size of 16, averaged over three runs after discarding the first three batches of each run as warm-up, with FPS reported as images processed per second. Floating-Point Operations Per second (FLOPs) were computed for a single (224×224×3) input using the ptflops library (version 0.7.5), reporting MACs directly as FLOPs (1 MAC = 1 FLOP). FLOPs could not be computed for Vim-B because its operations are unsupported by ptflops.

## Results and discussion

### Performance of individual models

A comparative evaluation of our models comprising CNN-, Transformer-, and Mamba-based backbones under the same experimental setting is presented in Table 3. To assess the stability of the reported metrics for a fixed trained model, we reported 95% bootstrap confidence intervals alongside each point estimate, taken as the 2.5th and 97.5th percentiles of the bootstrap distribution. These intervals were computed via 1000 bootstrap resamples of the test-set predictions, drawn with replacement and using a fixed random seed to ensure reproducibility. Overall, performance differences across architectural families were relatively small. This indicates that all three architectural families are equally suitable for retina-oriented eye disease classification.

**Table 3:** Performance comparison of CNN-, Transformer-, and Mamba-based backbones on the EDID.

Type	Model	Micro Accuracy	Macro Accuracy	F1 Score	AUC-ROC
CNN	RegNetY-16GF	0.7759 (0.7495 — 0.8013)	0.7329 (0.6859 — 0.7745)	0.7451 (0.7039 — 0.7778)	0.9600 (0.9477 — 0.9705)
	ConvNeXt-B	**0.7900** (0.7646 — 0.8155)	**0.7666** (0.7286 — 0.8057)	0.7523 (0.7161 — 0.7866)	0.9623 (0.9530 — 0.9702)
Transformer	DeiT-B	0.7382 (0.7100 — 0.7646)	0.7077 (0.6656 — 0.7483)	0.7030 (0.6645 — 0.7380)	0.9548 (0.9452 — 0.9643)
	SwinV2-B	0.7844 (0.7589 — 0.8098)	0.7560 (0.7146 — 0.7987)	0.7530 (0.7145 — 0.7859)	**0.9671** (0.9595 — 0.9738)
Mamba	Vim-B	0.7571 (0.7316 — 0.7825)	0.7284 (0.6866 — 0.7715)	0.7293 (0.6907 — 0.7662)	0.9571 (0.9470 — 0.9666)
	MambaVision-B	0.7844 (0.7589 — 0.8107)	0.7486 (0.7091 — 0.7884)	**0.7582** (0.7205 — 0.7902)	0.9647 (0.9578 — 0.9710)

Values in parentheses denote 95% bootstrap confidence intervals, obtained by resampling the test-set predictions of each model with replacement (1000 iterations). Best values are **bolded**, second best results are underlined.

Among the CNN-based models, ConvNeXt-B achieves the strongest performance. It also performs best across most metrics, obtaining the highest micro-accuracy (0.7900) and macro-accuracy (0.7666), along with a competitive F1 score (0.7523) and AUC-ROC (0.9623). It demonstrates improved performance over RegNetY-16GF, with higher micro- and macro-accuracy and F1 score. For Transformer-based models, SwinV2-B substantially outperforms DeiT-B on all reported metrics. In particular, SwinV2-B achieves the highest AUC-ROC (0.9671) among all evaluated models, while also attaining second-best results in micro-accuracy (0.7844), macro-accuracy (0.7560), and F1 score (0.7530). This indicates that hierarchical vision Transformers with shifted window attention provide more robust feature modeling than vanilla ViT. Within the Mamba-based models, MambaVision-B surpasses Vim-B across every metric. It also achieves the best overall F1 score (0.7582) across all the models, highlighting its strength in balancing precision and recall across classes. Its micro-accuracy (0.7844) matches that of SwinV2-B and is close to the top-performing ConvNeXt-B, while its AUC-ROC (0.9647) is the second highest overall.

These point estimates place ConvNeXt-B, SwinV2-B, and MambaVision-B ahead of the other three models; however, the bootstrap confidence intervals show that the gaps among them are narrow. The confidence intervals of these three models overlap substantially across all four metrics. The observed differences between them therefore fall within the range of test-set sampling variability and do not represent a definitive ranking. DeiT-B is the only model whose confidence intervals do not overlap with those of the top three across most metrics. Its lower performance holds up under resampling, since DeiT-B’s confidence interval does not overlap with those of the top three models, whereas every other pair of models does overlap. This means the gap between DeiT-B and the leading models is unlikely to be an artifact of which samples happened to fall in the test set, and more likely reflects a genuine difference in performance. RegNetY-16GF and Vim-B occupy an intermediate position. Their confidence intervals overlap both the top-performing cluster and, to a lesser extent, DeiT-B, so their relative standing is less conclusively established. These pairwise comparisons complement the McNemar significance testing reported below, which evaluates whether the differences in error patterns between models are statistically meaningful.

Taken together, ConvNeXt-B, MambaVision-B, and SwinV2-B form the strongest cluster of models, though their point estimates alone should not be read as a strict ranking. ConvNeXt-B attained the highest micro- and macro-accuracy. SwinV2-B attained the highest AUC-ROC and was also among the top two on the remaining three metrics. MambaVision-B attained the highest F1 score and was among the top two on micro-accuracy and AUC-ROC. Given this mixed pattern of performance, along with the overlapping bootstrap confidence intervals noted above, we treat these three models as roughly co-equal top performers rather than assigning a strict rank order among them.

### Computational efficiency analysis

Table 4 further compares the computational efficiency and training characteristics of the evaluated models. In general, models with stronger predictive performance tended to require higher computational resources. Vim-B is a notable exception: despite comparatively weak predictive performance, it required the longest training time and exhibited one of the highest levels of memory usage among all evaluated models. ConvNeXt-B achieved the best overall classification results, but this came at the cost of increased GPU memory usage and longer training time compared to more efficient models such as RegNetY-16GF and DeiT-B. Similarly, SwinV2-B delivered highly competitive performance, including the highest AUC-ROC among all evaluated architectures, although it also exhibited the highest memory consumption, together with lower inference throughput. In contrast, MambaVision-B demonstrated a more balanced trade-off between accuracy and efficiency. Despite maintaining competitive classification performance and achieving the highest overall F1 score, it required relatively low computational cost, low memory usage, faster training, and achieved the highest inference throughput among all models. Moreover, it substantially outperformed Vim-B in both predictive performance and runtime efficiency. Overall, these results reflect differences in how the evaluated models balance computational resources and predictive capacity, depending on their architectural design.

**Table 4:** Comparison of computational efficiency and training performance of CNN-, Transformer-, and Mamba-based vision models.

Architecture	Model	FLOPs	Peak GPU Memory	Training Time	Inference Throughput
		(G)	(MB)	Per Epoch (s)	(FPS)
CNN	RegNetY-16GF	16.01	8,205.5	43.62	471.7
	ConvNeXt-B	15.41	10,097.8	70.40	404.9
Transformer	DeiT-B	17.61	5,280.2	56.51	426.2
	SwinV2-B	10.55	11,483.6	74.99	268.0
Mamba	Vim-B	–	9,378.1	97.05	217.5
	MambaVision-B	15.00	7,087.0	46.16	496.5

The table reports floating point operations (FLOPs), peak GPU memory consumption during training, training time per epoch, and inference throughput measured in frames per second (FPS), all evaluated on an NVIDIA RTX 3090 GPU. Lower FLOPs, memory usage, and training time indicate better computational efficiency, while higher FPS reflect faster inference performance. Here, “–” indicates that the FLOPs value could not be computed.

### Significance testing

We performed pairwise McNemar tests [39] of significance with Holm–Bonferroni correction [40] between all models on the test set (see Fig 3). Our significance testing shows that RegNetY-16GF, ConvNeXt-B, SwinV2-B, and MambaVision-B do not differ significantly from one another in their error patterns despite differences in overall accuracy, yet all four significantly outperform DeiT-B. Although Vim-B was one of the worst-performing models, none of the comparisons involving it are statistically significant. This suggests that while Vim-B makes more errors overall, those errors are scattered across different samples rather than forming a consistent pattern shared with or distinct from any other model.

**Fig 3:**
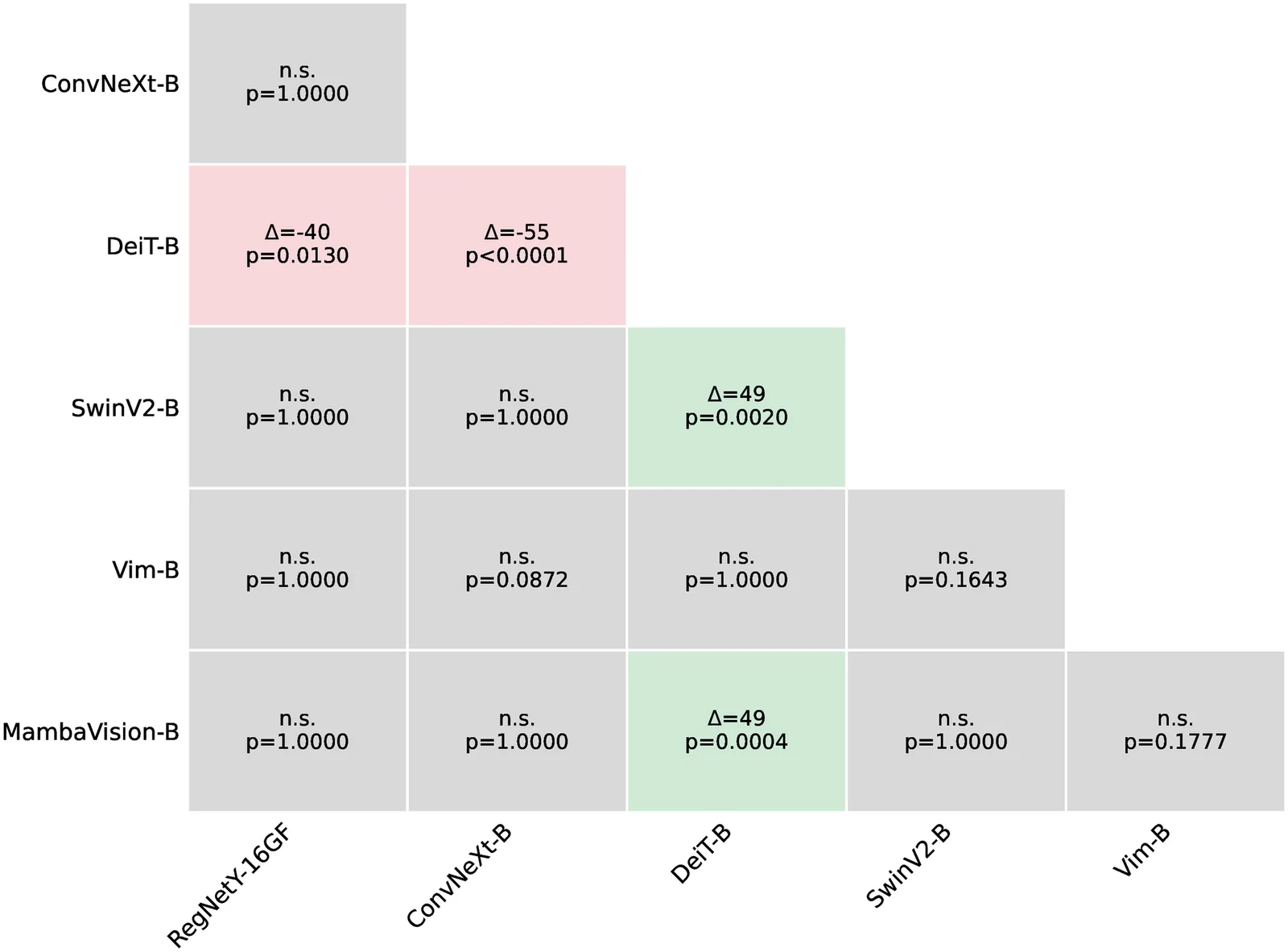
Pairwise McNemar significance comparison between all models on the test set. Each cell shows McNemar’s exact test with Holm-Bonferroni correction (α=0.05) comparing the row and column models. Here, ‘p’ indicates the p-value, Green indicates the row model makes significantly fewer errors, red indicates the column model makes fewer errors, and gray indicates no significant difference. In significant cells, Δ denotes the difference in discordant predictions.

### Performance of the ensemble model

We constructed an ensemble using the three best-performing individual models: ConvNeXt-B, MambaVision-B, and SwinV2-B, and evaluated it using two logit-level fusion strategies: mean fusion and max fusion. As shown in Table 5, the ensemble consistently outperforms individual models on most metrics, with the exception of Mean fusion’s macro-accuracy, which falls marginally short of ConvNeXt-B’s.

**Table 5:** Performance of logit-level ensembles of the top three models (ConvNeXt-B, MambaVision-B, and SwinV2-B) using mean and max fusion strategies.

Fusion Mode	Micro Accuracy	Macro Accuracy	F1 Score	AUC-ROC
Mean	0.7957	0.7644	0.7667	**0.9713**
Max	**0.7966**	**0.7777**	**0.7734**	0.9701

Best values are **bolded**.

Both fusion strategies demonstrate that combining predictions from multiple architectures yields more robust performance than any single model. Max fusion achieved the highest micro-accuracy (0.7966), macro-accuracy (0.7777), and F1 score (0.7734). It had particularly notable gains in macro-accuracy and F1 score, indicating improved class-balanced performance and a better precision–recall trade-off. Mean fusion, while slightly inferior in accuracy-based metrics, attained the best AUC-ROC, suggesting more calibrated predictions and stronger threshold-independent discriminative ability. Nevertheless, max fusion was found to be the preferable option, as its superior accuracy and F1 performance indicate a more effective preservation of strong class-wise predictions from individual models, which is critical for overall classification reliability.

### Class-wise performance

Table 6 presents a detailed class-wise comparison of ConvNeXt-B, MambaVision-B, SwinV2-B, and their ensemble (logit-level max fusion) across nine eye disease categories. Overall, DR and RD were consistently well recognized across all architectures, with all models achieving high accuracy and F1 scores (at or above 0.92). This consistency across architectures, including the ensemble, suggests that these diseases have salient and well-defined visual patterns that are effectively captured regardless of model design. Although DR had a substantially larger number of training samples than RD (1,056 versus 87 before oversampling), RD’s performance nearly matched DR’s, indicating that RD’s visual distinctiveness compensates for limited data. In contrast, CSCR and MS were the most challenging categories. Both of these classes exhibited low F1 scores across individual models, with only modest performance from the ensemble. The training set contained only 71 and 311 distinct images for CSCR and MS, respectively. The poor performance of CSCR can largely be attributed to this lack of training data. However, the limited image count does not fully explain the weak results for MS; this category was inherently difficult to learn. The ensemble improved MS detection compared with all individual models, although performance remains comparatively lower than for other classes, suggesting persistent feature ambiguity across architectures. Although the dataset contained a large number of healthy fundus images, performance on this category remained comparatively lower than for several disease classes, with most models achieving accuracies below 0.73. This may be due to the high variability in normal retinal appearance across patients and imaging conditions. The confusion matrices (Fig 4) show that healthy images are frequently misclassified as GLC, suggesting that normal optic disc variations, such as physiological cupping, may resemble early glaucomatous changes. In addition, unlike disease classes with distinct pathological markers, the healthy class lacks a clear positive visual signature, making it more difficult for the models to learn reliable decision boundaries. Performance on ODE and GLC was generally strong and stable across models, with F1 scores exceeding 0.75 in most cases. Although GLC had far more training images than ODE (944 vs. 89 distinct images), the limited data for ODE did not significantly affect performance. This suggests that ODE is inherently easier to classify or that the available images were highly representative of the class. SwinV2-B performs best on ODE, while ConvNeXt-B slightly outperforms the others on GLC. The ensemble delivers a balanced performance, reflecting the effective fusion of logits from complementary model architectures. MYA and RP showed greater variability among individual models, implying that limited training data may be hindering consistent learning. SwinV2-B performs best on MYA among the individual models, whereas MambaVision-B achieves the strongest RP F1 score, though ConvNeXt-B leads on RP accuracy. The ensemble achieves the highest F1 scores for both MYA and RP, indicating that logit-level fusion effectively combines complementary representations from different architectures. In contrast, RD performance remains identical across all models and the ensemble, suggesting that the class is already highly separable and gains little from fusion.

**Table 6:** Comparison of class-wise classification performance of ConvNeXt-B, MambaVision-B, SwinV2-B, and their ensemble.

Model	Metric	Class								
		Healthy	CSCR	DR	ODE	GLC	MS	MYA	RD	RP
ConvNeXt-B	Accuracy	0.6373	0.6500	0.9635	0.8000	0.8370	0.4831	0.6800	0.9200	0.9286
	Precision	0.8553	0.3824	0.9236	0.8000	0.6890	0.8958	0.6667	0.9200	0.7647
	F1 Score	0.7303	0.4815	0.9431	0.8000	0.7559	0.6277	0.6733	0.9200	0.8387
MambaVision-B	Accuracy	0.6618	0.5500	0.9402	0.8400	0.8259	0.5843	0.6300	0.9200	0.7857
	Precision	0.7849	0.5238	0.9497	0.7241	0.6737	0.7536	0.6632	0.9200	1.0000
	F1 Score	0.7181	0.5366	0.9449	0.7778	0.7421	0.6582	0.6462	0.9200	0.8800
SwinV2-B	Accuracy	0.7255	0.5500	0.9203	0.8800	0.7296	0.5955	0.8400	0.9200	0.6429
	Precision	0.8043	0.4400	0.9422	0.7857	0.7269	0.7571	0.5753	0.9200	0.9474
	F1 Score	0.7629	0.4889	0.9311	0.8302	0.7283	0.6667	0.6829	0.9200	0.7660
Ensemble	Accuracy	0.6814	0.6000	0.9402	0.8400	0.7926	0.6180	0.7500	0.9200	0.8571
	Precision	0.7989	0.4615	0.9497	0.7778	0.7086	0.7971	0.6522	0.9200	0.9231
	F1 Score	0.7354	0.5217	0.9449	0.8077	0.7483	0.6962	0.6977	0.9200	0.8889

**Fig 4:**
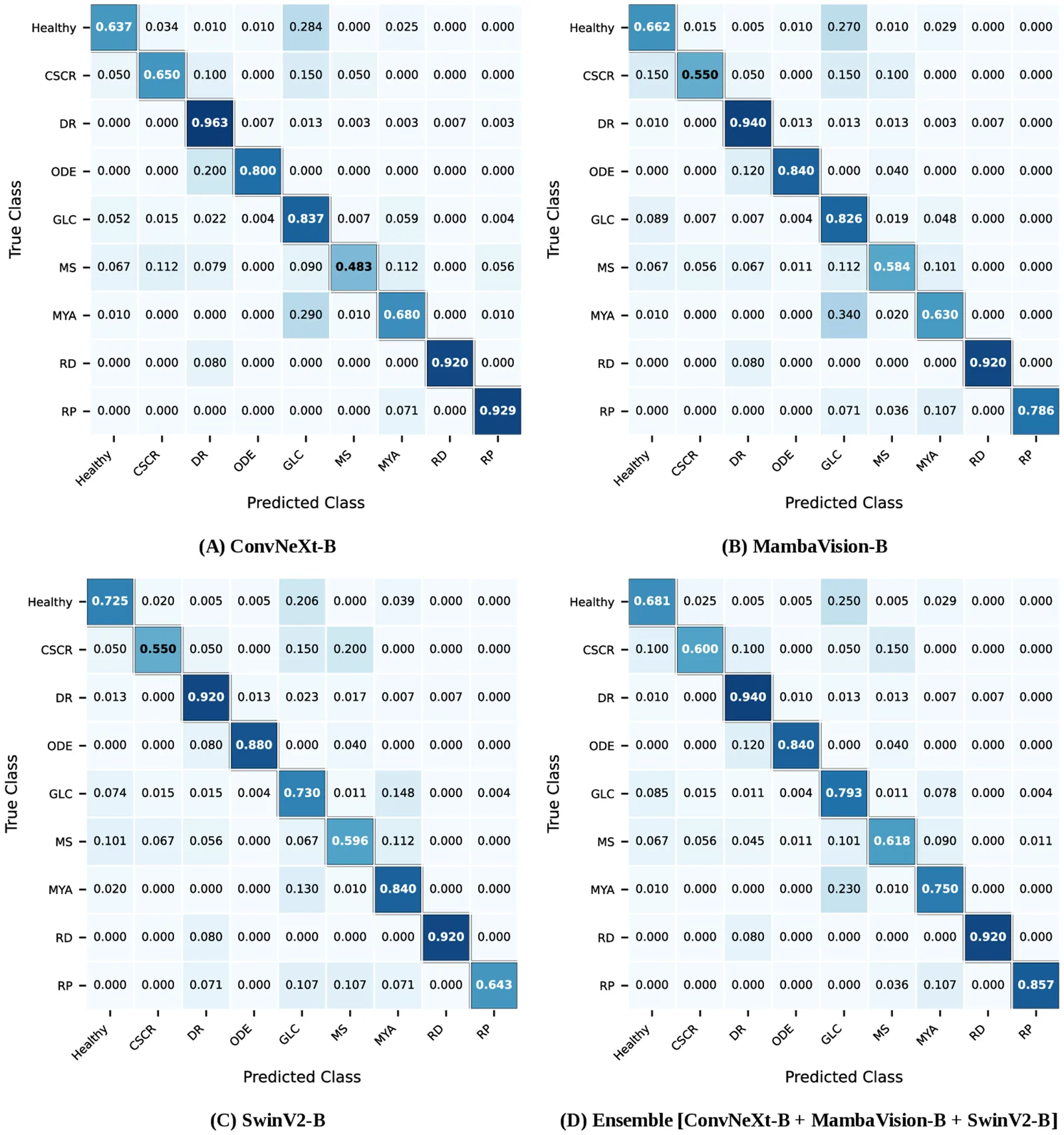
Confusion matrices showing class-wise prediction performance and error patterns. The results are presented for the top three best-performing models and their ensemble using logit-level max fusion. Here, (A) ConvNeXt-B, (B) MambaVision-B, (C) SwinV2-B, and (D) Ensemble.

A general examination of the confusion matrices in Fig 4 indicates that most errors were not random but concentrated among clinically or visually related classes, revealing consistent structural patterns in model misclassification behavior. Across models, the majority of errors clustered within a small subset of class pairs rather than being uniformly distributed. This suggests that the learned feature spaces of the models captured broad disease characteristics well, but sometimes struggled with defining inter-class boundaries. DR, ODE, GLC, RD, and RP achieved the highest classification accuracies across the three individual models as well as the ensemble, indicating that these conditions exhibit relatively distinctive visual characteristics. Healthy and MYA images were most frequently misclassified as GLC, likely because normal anatomical variation or myopic structural changes around the optic disc can resemble early glaucomatous alterations in cup-to-disc ratio and nerve fiber layer appearance. ODE was most often confused with DR, which may be attributed to overlapping signs such as vascular congestion, blurred disc margins, and secondary retinal changes that complicate clear separation in fundus imagery. CSCR was commonly misclassified as healthy, GLC, and MS, likely due to the subtle and variable presentation of serous retinal detachment, which in mild cases may appear nearly normal, while chronic CSCR can produce RPE alterations and peripapillary atrophy resembling glaucomatous structural changes or macular scarring. Misclassification rates for DR and GLC were low and remained relatively consistent across different disease grades. In contrast, MS exhibited a comparatively higher misclassification rate; however, these errors were not concentrated toward any single alternative class, but were instead distributed broadly across the remaining categories, indicating a lack of strong visual affinity with a specific competing condition. Overall, the findings suggest that most classification errors stem from overlapping structural and vascular characteristics among related retinal diseases, whereas conditions with more distinctive pathological signatures were identified consistently.

### Ablation study

To evaluate whether oversampling caused inflated minority-class performance, we conducted an ablation study using ConvNeXt-B under three settings: the original imbalanced split, class-weighted cross-entropy, and replication-based oversampling (Tables 7 and 8). If oversampling had caused memorization, the heavily replicated minority classes would be expected to achieve unusually high accuracy. However, CSCR and MS remained the most difficult classes under oversampling, with accuracies of 0.650 and 0.483, respectively, while weighted cross-entropy achieved higher scores on both classes without sample duplication. Oversampling also reduced performance on some majority classes, such as Healthy, indicating a redistribution of class sensitivity rather than artificial inflation. Overall, oversampling achieved the best micro-accuracy (0.7900), F1 score (0.7523), and AUC-ROC (0.9623), while weighted cross-entropy achieved the highest macro-accuracy (0.7739). However, it had the lowest overall micro-accuracy. These findings suggest that oversampling improved overall class balance and generalization performance, rather than artificially boosting minority-class results through memorization of duplicated samples. Therefore, oversampling was retained as the primary training strategy because it provided the best overall classification performance, as reflected by its higher micro-accuracy and F1 score. In contrast, weighted cross-entropy mainly improved macro-accuracy and the performance of two minority classes.

**Table 7:** Comparison of different class-balancing strategies using ConvNeXt-B under identical experimental conditions.

Method	Micro Accuracy	Macro Accuracy	F1 Score	AUC-ROC
Original Split	0.7721	0.7048	0.7272	0.9617
Weighted Crossentropy	0.7467	**0.7739**	0.7337	0.9595
Oversampling	**0.7900**	0.7666	**0.7523**	**0.9623**

**Table 8:** Class-wise accuracy comparison of different class-balancing strategies using ConvNeXt-B under identical experimental conditions.

Method	Healthy	CSCR	DR	ODE	GLC	MS	MYA	RD	RP
Original Split	**0.755**	0.400	0.894	0.800	0.822	**0.685**	0.460	0.920	0.607
Weighted Crossentropy	0.696	0.750	0.920	**0.840**	0.544	0.674	**0.870**	0.920	0.750
Oversampling	0.637	0.650	**0.964**	0.800	**0.837**	0.483	0.680	0.920	**0.929**

## Conclusion

In this study, we conducted a controlled comparative analysis of CNN-, Transformer-, and Mamba-based models for multi-class eye disease classification from fundus images. To the best of our knowledge, Mamba-based models have not previously been explored for this task. By employing consistent dataset splits and a unified training and evaluation pipeline, we addressed common methodological issues in prior studies, particularly those related to inconsistent validation practices and data leakage.

Our findings indicate that, on the EDID under controlled experimental conditions, no single model family demonstrated consistent superiority over the others. Instead, performance is strongly influenced by careful training design and rigorous data handling. The ensemble experiments further show that these architectural paradigms learn partially complementary feature representations, and that combining them can yield modest improvements in robustness and class-balanced performance. Class-wise analysis reveals that diseases with distinct anatomical or pathological patterns are detected more reliably, while conditions with subtle or overlapping visual characteristics remain challenging.

This study has some limitations. First, all experiments were conducted only on the EDID dataset, so the findings may not generalize well to retinal images from different clinical settings, imaging devices, or patient populations. Second, only six representative backbone architectures were evaluated in this study. Third, all models used the same classification head to maintain a fair comparison, which may not reflect the best possible performance of each architecture with customized designs. Fourth, all models were trained once due to computational constraints, so the reported results are based on single training runs. The bootstrap confidence intervals reported in this work capture test-set sampling variability under a fixed trained model; however, they do not capture run-to-run variability arising from different random initializations or data splits during training. Future work will focus on validating these findings on larger and more diverse multi-center retinal datasets. We also plan to include a wider range of CNN-, Transformer-, and Mamba-based architectures and explore backbone-specific classification head designs and optimization strategies to better understand their impact on retinal disease classification performance. In addition, we plan to conduct repeated training runs across multiple random seeds to quantify run-to-run variability.
